# Effectiveness of Learning through Play Plus (LTP Plus) Parenting Intervention on Behaviours of Young Children of Depressed Mothers: A Randomised Controlled Trial

**DOI:** 10.3390/children11060646

**Published:** 2024-05-27

**Authors:** Nusrat Husain, Rabia Sattar, Tayyeba Kiran, Mina Husain, Suleman Shakoor, Zamir Suhag, Zainab Zadeh, Siham Sikander, Nasim Chaudhry

**Affiliations:** 1 Division of Psychology and Mental Health, University of Manchester, Manchester M13 9PL, UK; 2 Mersey Care NHS Foundation Trust, Prescot L34 1PJ, UK; siham.sikander@liverpool.ac.uk; 3 Pakistan Institute of Living and Learning, Karachi 75600, Pakistan; rabia.sattar@pill.org.pk (R.S.); tayyaba.kiran@pill.org.pk (T.K.); suleman.shakoor@pill.org.pk (S.S.); zainab.zadeh@pill.org.pk (Z.Z.); nasim.chaudhry@pill.org.pk (N.C.); 4 Department of Psychiatry, University of Toronto, St. George Campus, 27 King’s College Circle, Toronto, ON M5S 1A1, Canada; mina.husain@camh.ca; 5 Centre for Addiction and Mental Health, 1001 Queen St. W., Toronto, ON M6J 1H4, Canada; 6 TVI-Trust for Vaccines and Immunization, Head Office, Al-Sehat Centre, Suite No 301, Rafiqui Shaheed Road, Karachi 75300, Pakistan; zamir.suhag@tvi.org.pk; 7 Department of Primary Care and Mental Health, University of Liverpool, Liverpool L69 3BX, UK

**Keywords:** child development, maternal depression, parenting intervention, Pakistan

## Abstract

Evidence has shown that parenting intervention programmes improve parental knowledge, attitudes, and practices, which helps in promoting child development. This study aims to examine the effectiveness of parenting intervention in improving child behaviours. This is a secondary analysis of data from a cluster-randomised controlled trial with depressed mothers aged 18–44 years with a child aged 0 to 36 months. This paper reports findings from the dataset of participants with a child aged between 24 and 36 months. Villages (*n* = 120) were randomised into either of two arms: learning through play plus (LTP Plus) or treatment as usual (TAU). LTP Plus is a 10-session, group parenting intervention integrated with cognitive behaviour therapy, delivered over 3 months. This secondary analysis reports findings on the Eyberg Child Behaviour Inventory (ECBI) and the Home Observation for Measurement of the Environment (HOME). Findings show a significant improvement in child behaviour (ECBI) scores (*p* < 0.011) and HOME scores (*p* < 0.001) in the intervention group compared to TAU at 3-month follow-up. In a low-resource setting, low-cost group parenting intervention delivered by community health workers has the potential to improve child behaviours and quality of the home environment. Parenting interventions aimed at improving child behavioural problems can have significant implications for the child, family, and broader societal outcomes. Addressing behavioural problems in early years, parenting interventions can potentially reduce long-term consequences and costs associated with untreated child behavioural issues.

## 1. Introduction

The early years of a child’s life are crucial for emotional regulation and attachment with the caregiver; however, maternal depression has been found to be a detrimental factor leading to emotional and behavioural problems in children [1,2,3]. Globally, 13% of women experience mental health problems after childbirth, primarily depression, the prevalence being higher in developing countries, i.e., 19.8% [4]. A recent meta-analysis shows that the prevalence of postnatal depression is found to be 30% in Pakistan [5].

Maternal depression may have an adverse impact on the home environment, including negative effects on the mother’s responsivity, acceptance of and involvement with the child, and decreased home environment functioning [6], all of which may lead to the child’s behavioural problems [7,8]. Home environment can have a significant effect on children’s social-emotional development [9], resulting in long-term negative consequences, including mental health problems and poor academic, occupational, and psychosocial functioning. Children of persistently depressed mothers are found more likely to have intense emotional and behavioural difficulties [3,10], highlighting the importance of early interventions [11,12]. A large part of the research evidence from LMICs is massively focused on the cognitive and physical development of young children [13,14,15]. However, there is a research gap in low and middle-income countries (LMICs) on social-emotional behaviours, particularly in young children not enrolled in school [9,16].

Country-level prevalence estimates show that over a quarter of preschoolers in Pakistan have low social-emotional skills, as indexed by aggressive behaviour, high levels of distraction, and poor social development [17]. Parental reports of children’s behavioural problems in LMICs are higher as compared to high-income countries, and male children are found more at risk for behavioural problems [11,18]. Evidence from Pakistan shows that children of depressed mothers experience externalising behaviour problems; however, maternal education, quality of the home environment, and social-emotional skills in the early years of the child’s life have the potential to promote healthy behavioural development [9]. Evidence shows that responsive parenting and cognitively stimulating learning activities can improve children’s cognitive, social-emotional, and physical development [19,20,21,22]. Research conducted in LMICs has demonstrated significant improvements in child development outcomes among vulnerable populations, through interventions encouraging nurturing care that includes stimulation, responsive caregiving, and learning opportunities in the home environment [16,20].

Parenting programmes designed to enhance positive parenting skills have proven effective in improving various aspects of child well-being, including physical, behavioural, cognitive, and emotional health [23,24,25,26,27]. Learning through play plus (LTP Plus) is a culturally adapted parenting programme focusing on stimulating early child development through parental involvement, learning, and attachment [25,28]. LTP Plus has been effective in improving maternal and child developmental outcomes. Globally, there have been recent developments in early parenting interventions to improve child physical, cognitive, and socio-emotional development; however, most of the research in LMICs is focused on improving child physical and cognitive development. Research on behavioural problems of children in LMICs is crucial for promoting child mental health, addressing socio-cultural challenges and disparities. Hence, there is a need to test the effectiveness of integrated parenting intervention in LMICs to improve child behavioural outcomes. The aim of this secondary analysis [25] was to explore the effectiveness of the LTP Plus parenting programme in improving child behaviour in a low-resource setting in Pakistan.

## 2. Materials and Methods

### 2.1. Study Design and Settings

This study is a secondary analysis of data from a mixed-method cluster-randomised controlled trial (cRCT) that tested the effectiveness of a manualised integrated parenting programme: learning through play plus (LTP Plus) for maternal depression. The study was conducted between January 2014 and June 2015 in low-resource settings of Gadap Town, Karachi, Pakistan. Gadap Town is one of the large towns of Karachi, Pakistan; it has 8 union councils (UCs) and 400 villages. The birth rate is around 15,000 births per year. As a low-resource setting with a high birth rate, Gadap Town is selected as a research site as per the need of the population. Villages were selected by consulting with local community leaders, taking into consideration the safety measures of the research team. Villages that were out of the trial catchment area and community leaders who did not agree to participate in the trial were excluded. The details of the methods and the main results of this trial have been reported elsewhere [25].

### 2.2. Recruitment and Randomisation

**Figure 1:**
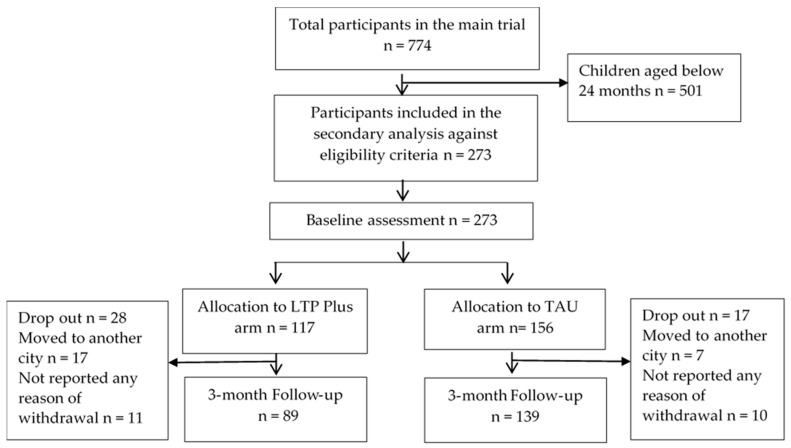
Consolidated Standards of Reporting Trials—CONSORT flow diagram.

The sample size for the main trial was derived from a previous cRCT in Pakistan [29] and was calculated based on an attrition rate of 10%, an effect size of approximately 0.2, and an intracluster correlation coefficient of 0.09. In the main trial, 774 depressed mothers from 120 villages were recruited. The inclusion criteria of mothers were 18–44 years old, with a child between 0 and 36 months old, having a diagnosis of major depressive episode on the Diagnostic and Statistical Manual of Mental Disorders, 4th Edition (DSM-IV), and an Edinburgh Postnatal Depression Scale (EPDS) score of more than 12. Mothers who were permanently resident in the trial catchment area, willing and able to provide informed consent and complete a baseline assessment were included. Exclusion criteria included any serious physical or mental health condition that may restrict participation in research, and active suicidal ideation. Villages were randomised into either of two trial arms: learning through play plus intervention (LTP Plus) or treatment as usual (TAU) arm. The secondary analysis of this study includes a sub-sample of 273 mother–child dyads, whose children were between the age of 24 and 36 months and who completed the Eyberg Child Behaviour Inventory (See Figure 1). For details on the participant flow and characteristics, see [25]. Written informed consent (thumb impression for those who were unable to read/write) was obtained from all participating mothers. Following informed consent, baseline assessments were carried out at participants’ homes. Randomisation of clusters (villages) was conducted by an off-site statistician using web software “http://www.randomisation.com (accessed on 31 January 2014)”. For the main trial, outcome assessments were completed at 3 months (end of intervention) and 6-months post-randomisation. However, the secondary analysis includes findings from baseline to 3-month outcome assessment. Researchers were blind to group allocation, and study participants were requested not to disclose their group status to the outcome assessors. The study was conducted in accordance with the principles of the Declaration of Helsinki. Ethics approval for the study was obtained from the ethics committee of Karachi Medical and Dental College (KMDC) (Ref #0019/13).

### 2.3. The Intervention: Learning through Play Plus (LTP Plus)

LTP Plus is a low-cost group parenting intervention, delivered by non-specialist Community Health Workers (CHWs), structured around a calendar to pictorially illustrate child developmental milestones. The calendar is devised for parents, depicting eight successive stages of child development from birth to 3 years through illustrations of parent–child play and other activities that promote parental involvement, learning, and attachment (see intervention details) [25]. For each developmental milestone, the activities have been divided into 5 areas of development including sense of self, physical development, relationships, understanding of the world, and communication. The group intervention was delivered by trained CHWs supported by master-level psychologists trained in LTP Plus, in 10 weekly sessions over a three-month period. There were up to 7 mothers in each intervention group. Each session lasted between 60 and 90 min.

### 2.4. Treatment as Usual (TAU)

Mothers in the TAU group received routine care from lady health workers, which may include any guidance related to maternal and child health. They were assessed at baseline and 3-month follow-up by researchers.

### 2.5. Outcome Assessment

#### 2.5.1. Child Outcomes

##### The Eyberg Child Behaviour Inventory (ECBI) [30]

The ECBI is a parent-reported behavioural rating scale that assesses child disruptive behaviours, and the extent to which parents find the behaviours troublesome. It is a 36-item questionnaire, developed for assessing children between the ages of 2 and 16 years. This measure is specifically focused on child behaviours that occur at home and consists of two subscales: (1) the intensity scale (where the parent indicates how often each behaviour currently occurs), and (2) the problem scale (where the parent indicates whether the identified behaviour is problematic). The individual intensity scores are aggregated, resulting in ranges of 36 to 252. The parents’ responses indicating a particular behaviour poses a problem for them (‘Yes’ = 1, ‘No’ = 0) are also summed for each item to generate the problem score, ranging from 0 to 36. Intensity scores exceeding 127 falls within the clinical range.

##### Home Observation for Measurement of the Environment (HOME) [31]

HOME is a parent report scale used to assess the child’s home environment, including the quality of cognitive stimulation and emotional support provided to the child by the family. A HOME interview is designed to be conducted in home settings with the child present and awake. This scale includes multi-response maternal reports and dichotomous observer ratings. It contains 45 items under 6 subscales, which are (1) emotional and verbal responsivity of the primary caregiver (items 1–11); (2) avoidance of restriction and punishment (items 12–19); (3) organisation of the physical and temporal environment (items 20–25); (4) provision of appropriate play materials (items 26–34); (5) parental involvement with the child (item 35–40); and (6) opportunity for variety in daily stimulation (items 41–45). Scores for each domain on the HOME inventory are obtained by averaging the responses to each question in that domain, resulting in a score ranging from 0 to 1, with higher scores indicating a more nurturing home environment. The scores within the lowest fourth spectrum of the score range indicate an environment that may pose a risk to some aspects of the child’s development [32]. The highest score for responsivity is 11, avoidance of restriction and punishment is 8, organization of the physical and temporal environment is 6, provision of appropriate play materials is 9, parental involvement with the child is 6, and opportunity for variety in daily stimulation is 5. All scales were translated into Urdu for the current study using the guidelines by [33] for translation and adaptation of scales.

### 2.6. Statistical Analysis

Data were analysed using SPSS software version 27.0. A two-sided significance level of 0.05 was used to determine the significance of the primary outcome (ECBI) and differences in secondary outcome variables. The demographic and other baseline variables were compared between study arms using descriptive statistics of means, standard deviations, and proportions. For the primary analyses, an independent sample t-test was used to compare the differences in the ECBI and HOME at a 3-month follow-up between the intervention and TAU arms. For the secondary analyses, analysis of covariance (ANCOVA) was used to compare groups, considering the baseline outcome values as a covariate.

## 3. Results

### 3.1. Participant Characteristics

The recruitment to the trial has been described in detail elsewhere [25]. The mean age of mothers in this sub-sample was 28.20 years (SD = 5.60). A total of 61.2% mothers had no formal education. Participants’ age, education, number of family members, household details, and income were recorded in socio-demographic form as these variables may have the potential to impact child developmental outcomes. Details of the sample are shown in Table 1.

### 3.2. Child Outcome

Results for child behaviour outcome revealed no significant difference in the Eyberg Child Behaviour Inventory (ECBI) scores between the groups at baseline (*p* = 0.285) (See Table 2). However, at the 3-month follow-up, there was a significant difference between the intervention and TAU group (<0.011), indicating that children of mothers in the intervention group had a significant decrease in behavioural problems as compared to the TAU arm. On the Home Observation for Measurement of the Environment (HOME) scale, at baseline, no significant difference was found on responsivity (*p* = 0.836), acceptance (*p* = 0.499), organization (*p* = 0.762), learning material (*p* = 0.835), and involvement (*p* = 0.240) between intervention and TAU groups (See Table 2). However, on the variety subscale, there was a significant difference in baseline scores (*p* = 0.020) in the intervention group compared to the TAU group. Results of the 3-month follow-up assessment demonstrated that the intervention group showed significantly higher scores on all these subscales compared to the TAU group (*p* < 0.001 for all). Overall, the intervention group demonstrated significant improvements in the HOME total score at the 3-month follow-up compared to the TAU group (*p* < 0.001), indicating the effectiveness of the intervention programme in reducing maternal depression, and hence enhancing the home environment.

## 4. Discussion

This was a secondary analysis of a mixed-method cluster-randomised controlled trial [25] conducted in a low-resource setting of Karachi, Pakistan. This study aimed to examine the effectiveness of the LTP Plus parenting intervention to improve maternal and child outcomes for depressed mothers of young children. The secondary analysis reported the effectiveness of the integrated parenting intervention in improving child behavioural problems.

Parental sensitivity toward their child has been proposed as an important mechanism for child development [34]. Evidence shows that maternal depression has been associated with lower quality of maternal bonding as well as with poor maternal sensitivity [35,36]. There is substantial evidence showing that parenting difficulties mediate the negative effect of postnatal depression on child development, with low maternal responsiveness leading to poor cognitive development [37,38], low sensitivity leading to insecure attachment [39], and poor support for infant emotional regulation leading to child behavioural problems [37]. These observations have led to the view that interventions for maternal depression might require a specific parenting component to benefit child outcomes. LTP Plus is a low-cost and low-literacy parenting intervention, culturally adapted for low-resource settings. LTP Plus promotes parental sensitivity, responsivity, attachment with the child, and parental involvement in child play and home environment, hence promoting the physical, cognitive, linguistic, and socio-emotional development of the child. Recent evidence shows that positive parenting and cognitively stimulating home environments have the potential to improve the developmental outcomes of children living in resource-constrained environments [9,20].

The impact of maternal depression on children has been emphasised [38], and problems such as insecure attachment, internalising and externalising problems, and cognitive difficulties have been reported as high in children of depressed mothers [40,41]. Evidence also shows that a broad range of negative child outcomes persist into late adolescence [38]. Findings of the current study show that there was a significant reduction in child disruptive behaviour scores on the Eyberg Child Behaviour Inventory at 3-month follow-up in the intervention group compared to TAU. These findings confirm that integrated parenting interventions not only help in reducing maternal depression, but it also has the potential to improve child behavioural outcomes. These findings are also confirmed by other research studies conducted in diverse settings showing that parenting interventions have strong evidence for enhancing parenting skills and in preventing emotional and behavioural problems in children [27,42,43,44,45].

A child’s home environment expands to include physical stimulation, nurture, and interaction with parents and the entire family system. Evidence shows that maternal depression may have an adverse and long-term impact on the home environment including negative effects on the mother’s acceptance of and involvement with her child, and decreased overall home environment functioning [6]. Evidence shows that maternal depression, parenting practices, and home environment have strong negative effects on a child’s internalising and externalising behaviour problems [7,8]. Findings of the secondary analysis of HOME scores demonstrated that there was a statistically significant improvement in the home environment of participants in the intervention arm, including improved parental responsivity, discipline and avoidance of punishment, enhanced organisation of environment, increased learning material, involvement with child, variety of activities, and stimulation compared to the TAU arm at 3-month follow-up. Evidence from other LMICs reported similar findings demonstrating that the participant receiving responsive stimulation had better HOME inventory scores on follow-up [46], and improved socioemotional developmental outcomes [47]. Evidence has shown that home environments in which children are raised can have long-lasting impacts on a range of developmental outcomes [8], including brain development [48], emotion regulation [49,50], and empathy [51], as well as on mental and physical health [52]. These findings have implications for young children as there is well-established evidence on the role of parental care that children receive being a key risk factor for behaviour problems [53]. Parenting interventions are effective in reducing behavioural problems; however, most programmes target preschool-aged and school-aged children [24,27]. Intervening earlier in childhood could be more effective from a clinical, economic, and educational perspective.

Parenting interventions tailored for low-resource settings to address child behavioural problems carry significant implications. This intervention has the potential to improve pathways to strengthen family bonds and enhance parenting skills, thereby fostering healthier child development trajectories. By equipping parents and caregivers with the tools to manage behavioural challenges effectively, these interventions can mitigate the risk of long-term negative outcomes such as academic underachievement, mental health issues, and involvement in risky behaviours. Parenting interventions targeting child behavioural issues are also substantially significant, impacting not only the child and family but also broader societal outcomes. By improving behavioural challenges early on, these interventions have the potential to mitigate the long-lasting repercussions and health care burden linked with unaddressed child behavioural issues. This low-cost parenting programme will help in scaling up the innovation across health services in Pakistan and LMICs and will provide the possible pathways to roll out the innovation at the national level through engagement with policymakers.

Strengths and Limitations: To our knowledge, this is the first study from Pakistan evaluating the effectiveness of an integrated parenting intervention to reduce disruptive behaviours in children. This study was conducted in a community setting, which strengthens the external validity of findings and generalisability to conditions in the real settings of LMICs. Further, research focusing on behavioural problems in children is highly focused on children enrolled in schools, but behavioural problems of children who are not school-going are under-researched and neglected. The current study provides insights into behavioural problems of children of mothers recruited from community settings. The trial used both self-reported and objective observational data to record the home environment through a standardised HOME inventory. As this was the secondary analysis of a cluster trial that had eligibility criteria to recruit mothers of children from birth until 3 years, the analysis included a small number of participants whose children were eligible for ECBI. Moreover, the trial did not assess the long-term impact of the intervention and demonstrate the long-term stability of positive intervention outcomes. LTP Plus has the potential to improve child behavioural outcomes by making the home environment cognitively responsive and stimulating. However, further studies in other populations and settings with longer follow-ups are warranted.

## 5. Conclusions

The integrated parenting intervention is not only effective in reducing maternal depression, it has the potential to improve parental knowledge, skills, and practices about child development. It is also effective in improving the home environment and child behavioural problems. Such findings highlight the importance of the quality of home environments and parenting practices on the behavioural development of children; this further emphasises the need for a family-centered approach to improve parenting practices and the home environment. LTP Plus is a low-cost, culturally adapted intervention that can be delivered effectively by trained CHWs using the task-shifting approach. The findings of this study also highlight the potential to scale up the LTP intervention to improve child behavioural outcomes.

## Figures and Tables

**Table 1:** Socio-demographic characteristics of the sample.

	TAU	Intervention	Total (n = 273)
	Mean (SD) and Median [IQR]		
Age mean (SD)	28.76 (4.97)	28.69 (5.73)	28.20 (5.60)
No. of family members	7 [6–10]	7 [5–11]	8 [6–12]
No. of children	3 [2–5]	3 [2–5]	3 [2–4]
Total monthly income	8000 [5000–12,000]	9000 [6000–12,000]	9000 [6000–15,000]
No. of rooms	1 [1–2]	1 [1–2]	1 [1–2]
	n (%)		
Education *			
Uneducated	79 (67.5%)	88 (56.4%)	167 (61.2%)
Up to primary	24 (20.5%)	41 (26.3%)	65 (23.8%)
Middle/matriculation	10 (8.5%)	21 (13.5%)	31 (11.4%)
Intermediate	4 (3.4%)	6 (3.8%)	10 (3.7%)
Family system			
Nuclear	66 (56.4%)	86 (55.1%)	152 (55.7%)
Joint **	51 (43.6%)	70 (44.9%)	121 (44.3%)
Status of house			
Ownership	106 (90.6%)	143 (91.7%)	249 (91.2%)
Rental	11 (9.4%)	13 (8.3%)	24 (8.8%)

* Primary (grade 5), middle (grades 6–8), matriculation (grades 9 and 10), and intermediate (grades 11 and 12). ** Joint family system (parents living with their children, parents, siblings, and grandparents).

**Table 2:** Comparative analysis of ECBI and HOME scales between TAU and intervention groups at two time points.

	*n*	TAU Mean (SD)	*n*	Intervention Mean (SD)	Mean Difference (95% CI)	ES	*p*-Value
ECBI *							
Baseline	117	17.91 (12.41)	156	16.35 (11.52)	1.56 (−1.31 to 4.43)	0.130	0.285
3rd month FU *	89	18.02 (11.90)	139	13.89 (11.77)	4.13 (0.97 to 7.29)	0.349	<0.011
Responsivity (HOME *)							
Baseline	117	7.54 (3.02)	156	7.46 (3.06)	0.08 (−0.66 to 0.81)	0.026	0.836
3rd month FU	116	7.73 (2.77)	156	9.55 (1.85)	−1.82 (−2.40 to −1.23)	0.747	<0.001
Acceptance (HOME)							
Baseline	117	3.50 (1.98)	156	3.66 (1.99)	−0.16 (−0.64 to 0.31)	0.081	0.499
3rd month FU	116	3.77 (2.32)	156	5.40 (1.67)	−1.63 (−2.13 to −1.13)	0.806	<0.001
Organization (HOME)							
Baseline	117	3.65 (1.63)	156	3.59 (1.61)	0.06 (−0.33 to 0.45)	0.037	0.762
3rd month FU	116	3.83 (1.66)	156	4.74 (1.23)	−0.91 (−1.28 to −0.56)	0.623	<0.001
Learning material (HOME)							
Baseline	117	1.92 (2.25)	156	1.98 (2.28)	−0.06 (−0.60 to 0.49)	0.026	0.835
3rd month FU	116	1.95 (2.28)	156	3.65 (2.45)	−1.70 (−2.28 to −1.13)	0.718	<0.001
Involvement (HOME)							
Baseline	117	2.76 (1.87)	156	3.02 (1.74)	−0.26 (−0.69 to −0.17)	0.144	0.240
3rd month FU	116	3.64 (1.98)	156	4.74 (1.72)	−1.10 (−1.56 to −0.65)	0.593	<0.001
Variety (HOME)							
Baseline	117	2.60 (1.12)	156	2.91 (1.07)	−0.31 (−0.58 to −0.05)	0.283	0.020
3rd month FU	116	3.01 (1.08)	156	3.64 (0.92)	−0.63 (−0.87 to −0.39)	0.628	<0.001
HOME Total							
Baseline	117	21.97 (7.91)	156	22.62 (7.53)	−0.66 (−2.51 to 1.20)	0.084	0.486
3rd month FU	116	23.92 (8.56)	156	31.73 (6.26)	−7.81 (−9.66 to −5.96)	1.042	<0.001

* ECBI: Eyberg Child Behaviour Inventory. FU: Follow-up. HOME: Home Observation for Measurement of the Environment.

## Data Availability

Requests for sharing the anonymised data should be addressed to the lead author. Data is not publicly available due to ethical reasons.
